# Nucleostemin Knockdown Sensitizes Hepatocellular Carcinoma Cells to Ultraviolet and Serum Starvation-Induced Apoptosis

**DOI:** 10.1371/journal.pone.0141678

**Published:** 2015-10-30

**Authors:** Fuwen Yuan, Qian Cheng, Guodong Li, Tanjun Tong

**Affiliations:** 1 Research Center on Aging, Department of Biochemistry and Molecular Biology, School of Basic Medical Sciences, Peking University, Beijing, China; 2 Beijing Key Laboratory of Protein Posttranslational Modifications and Cell Function, Beijing, China; University of Illinois at Chicago, UNITED STATES

## Abstract

Nucleostemin (NS) is a GTP-binding protein that is predominantly expressed in embryonic and adult stem cells but not in terminally differentiated cells. NS plays an essential role in maintaining the continuous proliferation of stem cells and some types of cancer cells. However, the role of NS in hepatocellular carcinoma (HCC) remains unclear. Therefore, this study aimed to clarify the role of NS in HCC. First, we demonstrated high expression of NS in most HCC cell lines and liver cancer tissues. NS knockdown induced a severe decline in cell viability of MHCC97H cells as detected by MTT and cell proliferation assays. Next, we used ultraviolet (UV) and serum starvation-induced apoptosis models to investigate whether NS suppression or up-regulation affects HCC cell apoptosis. After UV treatment or serum starvation, apoptosis was strongly enhanced in MHCC97H and Bel7402 cells transfected with small interfering RNA against NS, whereas NS overexpression inhibited UV- and serum-induced apoptosis of HCC cells. Furthermore, after UV irradiation, inhibition of NS increased the expression of pro-apoptosis protein caspase 3 and decreased the expression of anti-apoptosis protein Bcl-2. A caspase 3 inhibitor could obviously prevent NS knockdown-induced apoptosis. In conclusion, our study demonstrated overexpression of NS in most HCC tissues compared with their matched surrounding tissues, and silencing NS promoted UV- and serum starvation-induced apoptosis of MHCC97H and Bel7402 cells. Therefore, the NS gene might be a potential therapeutic target of HCC.

## Introduction

Nucleostemin (NS), also named guanine nucleotide binding protein-like 3 (GNL3), is a nucleolar protein. Mammalian NS was first cloned from neural stem cells [1]. Later studies reported that NS is also abundantly expressed in other types of stem cells such as embryonic and mesenchymal stem cells as well as several types of cancer cells and adult testes [2–6]. The vertebrate NS family includes NS, GNL3, and Ngp-1, all of which contain a unique MMR1-HSR1 domain of five GTP-binding motifs arranged in a circularly permuted order [6]. Certain molecules regulate the partitioning of NS between the nucleolus and nucleoplasm, such as GTP and cellular senescence-inhibited gene (CSIG) [1, 7]. NS protein complex shuttling between the nucleolus and nucleoplasm might play an important role in cell proliferation and apoptosis.

As a nucleolar protein, NS not only has critical roles in pre-RNA processing [8], but also many other functions such as regulation of cell growth and cell cycle progression [9, 10, 11]. First, as a direct transcriptional target of the oncoprotein c-Myc, NS functions downstream of Myc as a rate-limiting regulator of cell proliferation and transformation, which is independent of its putative role in the p53 pathway [12]. Furthermore, NS regulates the cell cycle by binding to certain proteins implicated in cell cycle control, including p53, murine double minute 2 (MDM2), and nucleophosmin [1, 13–15]. In most cell lines, NS knockdown causes G0/G1 arrest, whereas in others, G2/M arrest is observed after NS knockdown [14–17]. In addition, NS can delay cellular senescence through negative regulation of telomeric repeat binding factor 1 (TRF1) protein stability by a direct interaction with TRF1 to prevent its dimerization or by promotion of PML-IV recruitment to SUMOylated TRF1 [18–19]. A recent study even showed that depletion of NS in cultured neural stem cells triggers replication-dependent DNA damage and perturbs self-renewal by direct recruitment to sites of DNA damage [20]. NS also participants in the apoptosis of cancer cells, mainly in a p53-dependent manner [21–24]. Knockdown of NS in PC3 cells, a human prostate cancer cell line, increases the expression of apoptotic related genes [23]. On the other hand, no enhancement of apoptosis is found in NS-mutant mouse embryos [25].

Numerous studies demonstrate that NS regulates the proliferation and apoptosis of cancer cells. However, there are very few studies on the expression and functions of NS in hepatocellular carcinoma (HCC). This study aimed to examine the expression of NS in a series of HCC cell lines and tissues, and investigate its function in HCC cell apoptosis.

## Materials and Methods

### Cell culture

The human immortalized hepatocyte cell line L02 and HCC cell line Huh7 were purchased from China Center for Type Culture Collection (CCTCC, Wu Han, China). MHCC97L, MHCC97H, and SMMC7721 cells have been described previously [26, 27]. Bel7402 and HepG2 cells were obtained from Prof. Li [28]. Bel7402, MHCC97H, MHCC97L and HepG2 cells were grown in DMEM with 10% fetal bovine serum. L02 and SMMC7721 cells were cultured in RPMI-1640 medium supplemented with 10% FBS. All cells were incubated at 37°C with 5% CO2.

### Patients and surgical specimens

Cancer tissues and para-cancerous tissues of patients 1–10 were collected from Henan Cancer Hospital, also named Affiliated Oncology Hospital of Zhengzhou University, and have been provided by Prof. Lu [29]. Other clinical specimens were obtained from the Tissue Bank at the Peking University School of Oncology and have been described in our previously study [30, 31].This study was approved by the Ethics Committee of Peking University Health Science Center. Informed consent was obtained from each participant. The patients comprised 12 men and 6 women with an average age of 63 years. The detailed information of the 18 patients is described as Table 1.

**Table 1:** Clincopathological Characteristics.

Clincopathological Characteristics		Male	Female
**Total**		12	6
**Age**	≤50	10	4
	>50	2	2
**Serum AFP**	Positive	3	4
	Negative	9	2
**Tumor Grade**	G1	1	0
	G2	5	2
	G3	3	2
	Unknown	3	2

### Cell culture

HCC cell lines, including Bel7402, MHCC 97H, MHCC 97L, Huh7 and HepG2, were cultured in Dulbecco’s modified Eagle’s medium (DMEM) supplemented with 10% fetal bovine serum (FBS). L02 (a human immortalized hepatocyte cell line) and SMMC7721 cells were cultured in RPMI 1640 medium containing 10% FBS. All cells were incubated at 37°C with 5% CO2.

### Plasmids and small interfering RNA preparations

Full-length NS cDNA was amplified from 293 cells by polymerase chain reaction (PCR) using FLAG-tagged primers at the N-terminus, which was inserted between the BamHI and XhoI sites of the pCMV vector (Clontech, Mountain View, CA, USA). Small interfering (si)RNA targeting NS (siNS) was 5′-CCAUUCGGGUUGGAGUAAU-3′ and negative control siRNA (siNC) was 5′-UUCUCCGAACGUGUCACGUTT-3′. RNA interference for 48 or 72 h in HCC cells was carried out according to the manufacturer’s protocol using Lipofectamine 2000 (Invitrogen).

### RNA isolation and quantitative reverse-transcription (RT)-PCR

Quantitative RT-PCR was performed with an ABI PRISM 7500 Sequence Detection System (Applied Biosystems, Foster City, CA, USA) using SYBR Green PCR master mix (Applied Biosystems). Primer sequences were 5′-ATGACCTGCCATAAGCGGTAT-3′ and 5′-ACCATTGAGAGCTACTGTCAGG-3′ for NS, and 5′-GTAACCCGTTGAACCCCATT-3′ and 3′- CCATCCAATCGGTAGTAGCG-5′ for 18sRNA. Relative quantification was conducted using the comparative cycle threshold method.

### Western blotting

The protocol for western blotting has been described in a previous study [32]. Antibodies included anti-NS (1:2000; Epitomics and Abcam), anti-human p27 (1:2000; MBL), anti-skp2 (1:1000, Epitomics), anti-glyceraldehyde-3-phosphate dehydrogenase (GAPDH) (1:5000, Epitomics), and anti-p53 (1:1500, Anbo).

### Cell cycle analysis

First, MHCC97H cells were transfected with siNC or siNS for 48 h and then harvested and fixed in 70% ethanol. Cells were washed twice with phosphate-buffered saline and then incubated with 50 mg/mL RNaseA for 30 min at 37°C, followed by addition of 2.5 mg/mL propidium iodide. Quantification of the percentage of cells in specific cell cycle phases was performed by flow cytometric analysis using a FACScan flow cytometer.

### Apoptosis analysis

Cells were plated on 60- or 100-mm culture dishes. For ultraviolet (UV) irradiation, the culture medium was removed and the culture dishes were placed uncovered in a UV cross-linker (model UVC-500; Hoefer, Holliston, MA, USA). Cells were irradiated at 25–200 J/m2 UV-C in the initial dose response analysis, and then 50 J/m2 UV-C was used for experiments. Immediately after irradiation, medium was added to the cells, followed by culture for the appropriate time. For serum starvation, the culture medium was removed and DMEM without FBS was added to the cells, followed by further culture for 24 h. Cells were harvested and centrifuged at 1500 rpm for 5 min at room temperature. The medium was removed, and the cells were washed once in phosphate-buffered saline. Cells were then resuspended in cold binding buffer, followed by addition of annexin V-FITC and propidium iodide. The cells were incubated at room temperature for 15 min in the dark and then analyzed by flow cytometry.

### Analysis of apoptosis in the presence of a caspase 3 inhibitor

Briefly, MHCC97H and Bel7402 cells plated in 6-cm dishes were transfected with siNC or siNS. After transfection for 6 h, the cells were cultured in the presence or absence of a caspase 3 inhibitor (10 μM) for 48 h. The cell permeable inhibitor for caspase 3 was purchased from Santa Cruz Biotechnology (sc-3075). Cells were harvested, and percentages of apoptotic cells were analyzed by flow cytometry.

### Statistical analysis

Baseline data of patients are expressed as means ± standard deviation (SD) or median values from at least three independently performed experiments. Statistical analysis was performed using SPSS 20.0 for Windows. The Student’s t-test was used to analyze statistical differences between groups under various conditions. A two-tailed P-value of less than 0.05 was considered significant.

## Results

### HCC is associated with increased expression of NS

**Fig 1:**
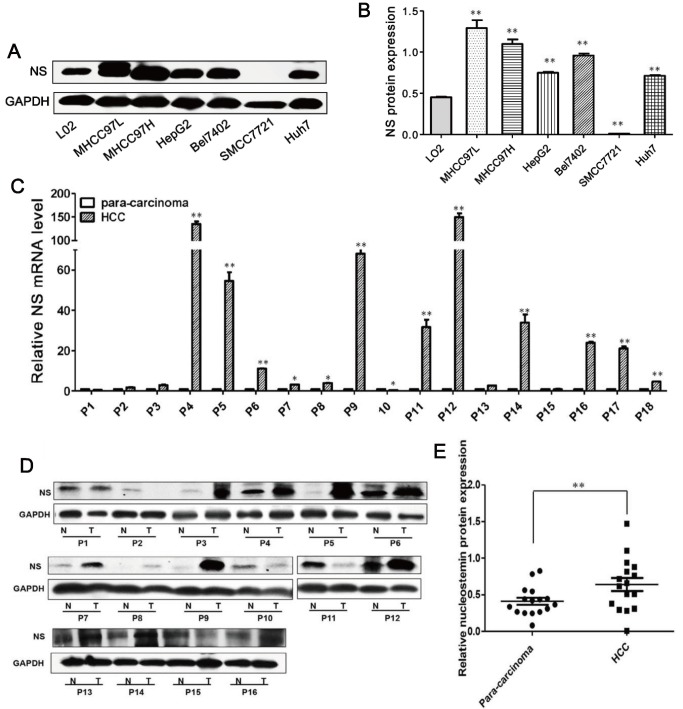
Expression of NS in HCC cell lines and tissues. (A) Western blot analysis of NS protein expression in HCC cells and the hepatic non-tumor cell line L02. (B) Quantification of NS protein levels in various HCC cell lines and L02 cells. Image J method was performed to quantificate the western blots (n = 3), and GAPDH is a loading control. *P<0.05, **P<0.01. (C) Expression of NS mRNA in 18 matched samples of liver tumor and para-cancerous tissues. (D) Representative western blot of NS in HCC and para-cancerous tissues. N, para-cancerous tissue; T, tumor. (E) NS protein expression in 16 paired samples of and HCC and para-carcinoma tissues. **P<0.01.

In this study, western blot assays were carried out to investigate the expression of NS protein in HCC cell lines from various sources as well as an immortalized liver cell line, L02. The expression of NS protein in most HCC cell lines including MHCC 97H, MHCC 97L, HepG2, Bel7402, and Huh7 cell lines was higher than that in the non-cancerous liver cell line L02 (Fig 1A and 1B). The migration ability of MHCC97H is higher than SMMC7721 cell line. And NS protein levels of MHCC97H is higher than SMMC7721 cells. MTT assays also indicated that HepG2, MHCC97H, SMMC7721, Bel7402 HCC cell lines had higher growth rates compared with the L02 cell line (S1 Fig). The result suggested that NS might play an important role in HCC cells. However, the NS protein levels were not completely correlate with proliferation rates for these cell lines. The expression of NS may be affected by many factors including tumor migration, tumor proliferation or the genetic background of cell lines.

NS has critical roles in cell proliferation and apoptosis, and high expression in cancer cells and tissues such as gastric cancer, leukemia, HeLa cells [3, 22, 24, 25]. Next, real-time PCR and western blot assays were performed to detect the expression of NS mRNA and protein in 18 matched pairs of HCC samples. NS mRNA levels were increased in 15/18 of HCC samples, compared with adjacent non-tumor tissue, and decreased in 3/18 of HCC samples (Fig 1C). The relative expression of NS protein was 1.73-fold, in HCC tissues compared with surrounding non-tumor tissues (Fig 1D and 1E). The process that NS mRNA was translated to NS protein might be regulated, so discrepancy between nucleostemin mRNA and protein levels in patient samples 3 and 11 was observed.

### Silencing of NS suppresses MHCC97H cell viability

**Fig 2:**
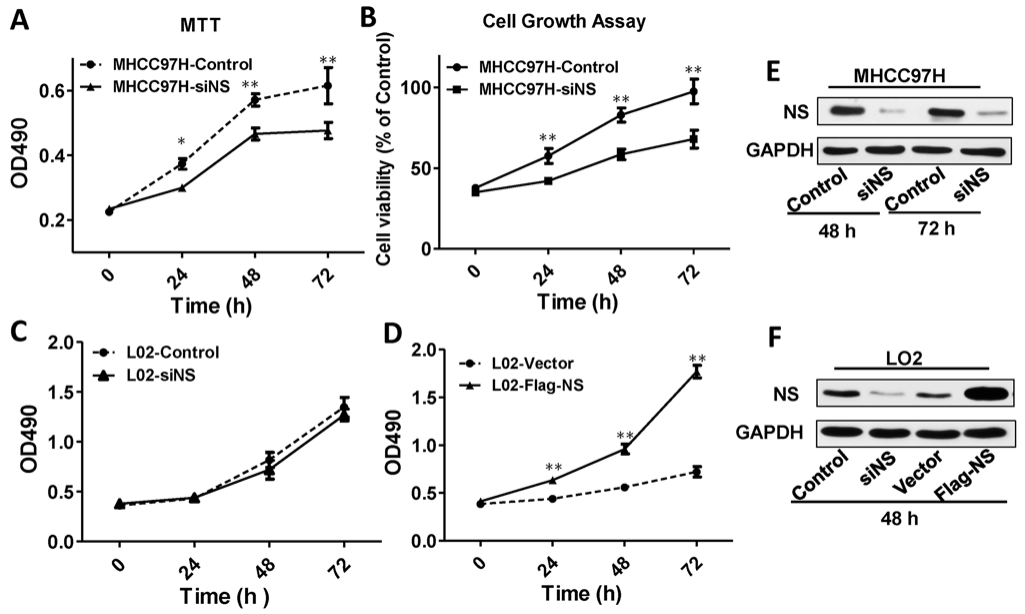
Knockdown of NS suppresses MHCC97H cell proliferation. (A) MTT assays of MHCC97H cells treated with siNC or siNS at 1, 2, and 3 days. Each value represents the average of three independent experiments, and bars indicate the standard error of the mean. *P<0.05, **P < 0.01. (B) Cell growth curves of MHCC97H cells were constructed by measuring cell proliferation every 12 h after transfection with siNS-and siNC for 24 h. (C and D) MTT assays of L02 cells treated with siNS or overexpressing NS. Each value represents the average of four independent experiments, and bars indicate the standard error of the mean. **P<0.01. (E) Knockdown of NS in MHCC97H cells by siRNA transfection at 48 and 72 h. Western blot analysis was performed using antibodies against NS and GAPDH as the loading control. (F)Western blotting assays of transient transfection of NS siRNA or overexpressed plasmids in L02 cells.

Our results demonstrated that NS was preferentially expressed in most HCC cell lines and tissues, suggesting that NS plays an important role in the proliferation or apoptosis of HCC cells. To test this hypothesis, we performed MTT and cell proliferation assays. MTT assays showed repression of cell growth after NS knockdown for 48 h (Fig 2A), and the cell proliferation assay confirmed this result (Fig 2B). In immortalized hepatocyte L02 cells, NS knockdown resulted in slight growth repression (Fig 2C), which might have been due to low background expression. On the other hand, NS overexpression obviously promoted L02 cell proliferation (Fig 2D). The expression of L02 is lower than other tumor cells, so knockdown of NS in L02 cells could not inhibit cell proliferation significantly. To verify the knockdown or overexprssion efficiency of siNS, western blotting was carried out at 48 or 72 h after transfection, which confirmed a significant reduction in the protein level of NS (Fig 2E and 2F). In SMCC7721 cells, transfection with siNS or NS overexpression had no significant effect on cell proliferation (S2 Fig). The SMCC7721 is a NS-null cell line, so some other factors might have more important effect on SMMC7721 cell proliferation rather than NS.

### Knockdown of NS induces cell cycle arrest in G2/M phase slightly

**Fig 3:**
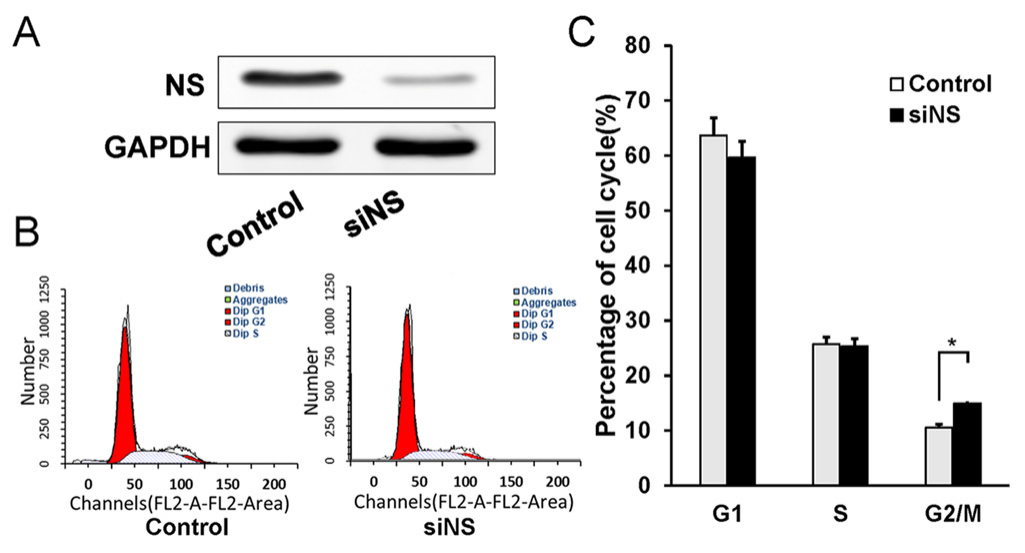
Effect of NS down-regulation on cell cycle progression in MHCC97H cells. The cell cycle distribution was examined by flow cytometric analysis. (A) Western blot analysis siNS-treated cells showed a dramatic reduction in the expression of NS after transient transfection at 48 h. (B) Knockdown of NS by RNA interference in MHCC97H cells induced cell cycle arrest in G2/M phase at 48 h after transfection. (C) Quantitative analysis of the cell cycle distribution at 48 h. Each value represents the average of three independent experiments, and bars indicate the standard error of the mean. **P<0.01.

To further examine the suppressive effects of siNS on MHCC97H cell growth, we performed cell cycle distribution analysis by flow cytometry at 48 h after siNS transfection. As shown in Fig 3, NS knockdown induced cell cycle arrest in G2/M phase slightly in MHCC97H cells. Compared with the siNC group, the percentage of cells in G2/M phase of the siNS group was increased by 4.8% at 48 h (Fig 3, P<0.01), while no change was found in the ratio of cells in G1 and S phases. These results demonstrate that NS silencing may induce cell cycle arrest at G2/M phase.

### NS inhibition induces apoptosis of HCC cells

**Fig 4:**
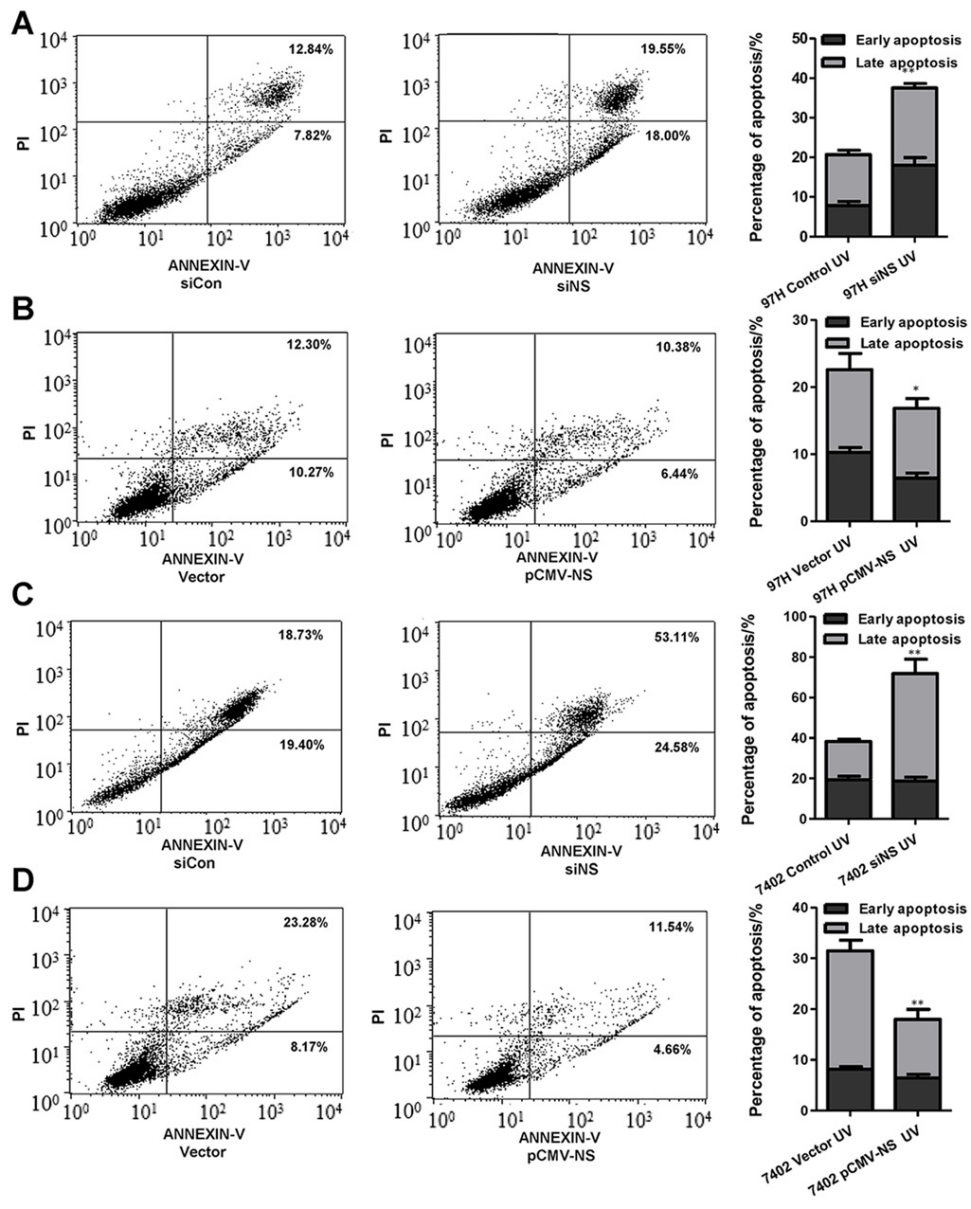
UV-induced apoptosis is regulated by NS in MHCC97H and Bel7402 cell lines. (A) UV irradiation-induced apoptosis in MHCC97H-control and MHCC97H-siNS cells. The cells were plated on 60- or 100-mm culture dishes, and siRNA or plasmids were introduced using Lipofectamine 2000 according to the manufacturer’s protocol. For UV irradiation, the culture medium was removed after transfection for 24 h, and then the culture dishes were uncovered and placed in a UV cross-linker for the appropriate times. (B) Apoptosis distributions of MHCC97H-vector and MHCC97H-NS cells after UV irradiation. (C) UV irradiation-induced apoptosis in Bel7402-control and Bel7402-siNS cells. (D) Apoptosis distributions of MHCC97H-vector and MHCC97H-NS cells after UV irradiation. All apoptosis quantification was displayed as mean ± SD values. Each treatment was repeated in triplicate with NS knockdown or overexpression was visibly higher than that of control group. **P<0.01.

To investigate the role of NS in UV-induced apoptosis of HCC cells, we performed flow cytometric analysis of cellular apoptosis following NS knockdown or overexpression in HCC cells. Our results showed that the percentage of apoptotic MHCC97H cells after NS knockdown was significantly higher than that of the control following irradiation with 50 J/m2 UV-C (37.55±1.82% vs. 20.66±2.05%), whereas overexpression of NS obviously suppressed the UV-induced apoptosis (Fig 4A and 4B; P<0.01). A similar result was obtained with the Bel7402 cell line (Fig 4C and 4D). The percentage of apoptotic cells was 77.69±3.13% after NS knockdown, which was higher compared with the control. On the other hand, the percentage of apoptotic cells was reduced about 5% with NS overexpression compared with the siNC group (Fig 4C and 4D; P<0.01). Furthermore, NS knockdown promoted serum starvation-induced apoptosis (S3 Fig).

### Knockdown of NS affects the expression of cleaved caspase 3 and Bcl-2

**Fig 5:**
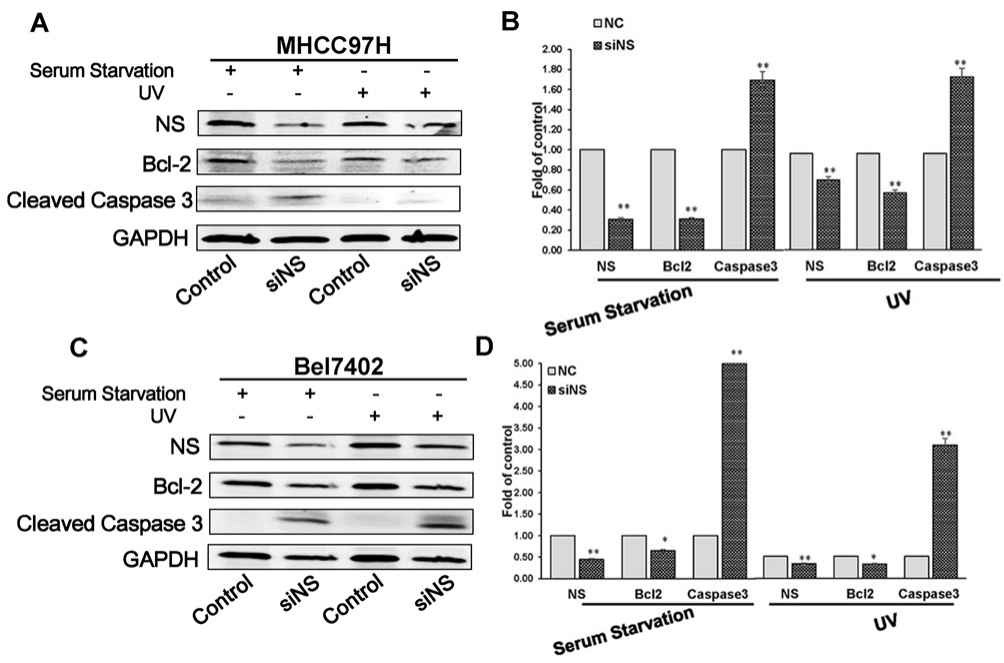
Knockdown of NS affects the expression of cleaved caspase 3 and Bcl-2. (A) Changes in the expression pattern of certain apoptosis regulatory genes in siNS-treated MHCC79H cells. For UV irradiation, the culture medium was removed after transfection for 24 h. The culture dishes were then uncovered and placed in a UV cross-linker, and the cells were cultured for the appropriate times. For serum starvation, the culture medium was replaced with DMEM without FBS, followed by 24 h of culture. Total proteins were extracted for western blot analysis. Expression of pro-apoptotic caspase 3 was higher than in the control after irradiation with 50 J/m2 UV-C or serum starvation for 24 h, while expression the anti-apoptotic protein Bcl-2 was lower. (B) Quantification of NS, caspase 3, and Bcl-2 protein levels in MHCC97H cell lines. Image J analysis was performed to quantificate the western blots (n = 3), and GAPDH is a loading control. *P<0.05, **P<0.01. (C) After UV-induction or serum starvation, changes in the expression pattern of certain apoptosis regulatory genes in siNS-treated Bel7402 cells. (D) Quantification of NS, caspase 3, and Bcl-2 protein levels in Bel7402 cell lines. Image J analysis was performed to quantificate the western blots (n = 3), and GAPDH is a loading control. *P<0.05, **P<0.01.

To further explore the mechanism promoting apoptosis by NS silencing, western blot assays were performed to examine the protein levels of cleaved caspase 3 and Bcl2. Expression of pro-apoptotic protein caspase 3 was higher in the MHCC97H-siNS group than the control after irradiation with 50 J/m2 UV-C or serum starvation for 24 h, while the level of anti-apoptotic protein Bcl-2 in the MHCC97H-siNS group was lower than that in the control group (Fig 5A and 5B). Similar results were obtained in Bel7402 cells (Fig 5C and 5D). Taken together, these result suggested that silencing NS promoted the apoptosis of MHCC 97H and Bel7402 cells after UV irradiation or serum starvation.

### Inhibition of caspase 3 prevents apoptosis induced by UV with knockdown of NS

**Fig 6:**
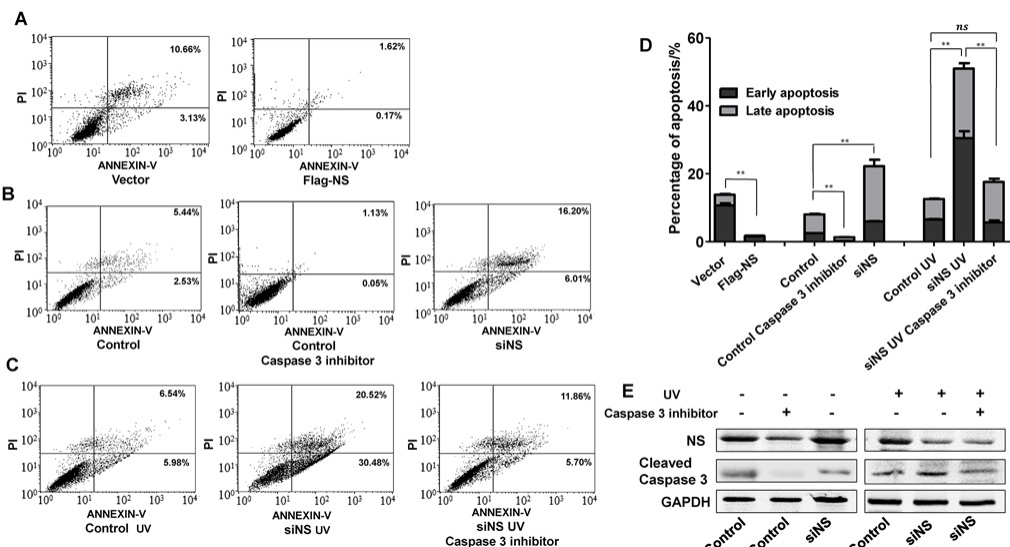
Caspase 3 inhibition prevents apoptosis induced by NS knockdown. (A) Flow cytometric analysis of apoptosis after transfection with Flag-NS or the control vector. (B) Apoptosis distributions of MHCC97H cells after transfection with siNS or siNC at 48h and MHCC97H-siNC cells treated with the caspase 3 inhibitor. FITC-labeled annexin V-positive cells (upper and lower right) were considered as apoptotic cells. (C) Apoptosis distribution induced by UV irradiation in MHCC97H-siNC and MHCC97H-siNS cells cultured in the presence or absence of the caspase 3 inhibitor (10 μM) for 48 h. FITC-labeled annexin V-positive cells (upper and lower right) were considered as apoptotic cells. (D) Quantitative analysis of the percentages of apoptotic MHCC97H cells cultured in the presence or absence of the caspase 3 inhibitor (10 μM) for 48 h. Apoptosis is displayed as mean ± SD values. Each treatment was repeated in triplicate with NS knockdown or overexpression was visibly higher than that of control group. **P<0.01. (E) Western blotting was carried to confirm inhibition of caspase 3 by the caspase 3 inhibitor.

NS overexpression suppressed UV-induced apoptosis slightly, however, compared with the control, our results showed that NS suppression induced apoptosis in about 15% of cells, (Fig 6A and 6B). These data suggested that NS knockdown might play a more important role in UV-induced apoptosis. Caspase 3 is an important factor in apoptosis. To further clarify that silencing NS induced apoptosis through caspase 3 activation, we analyzed the UV-induced apoptosis of MHCC97H-siNS cells in the presence of a caspase 3 inhibitor. After transfection of siNC or siNS for 6 h, MHCC97H cells were cultured in the presence or absence of the caspase 3 inhibitor (10 μM) for 48 h and then treated with 50 J/m2 UV-C. The percentages of apoptotic cells were analyzed by flow cytometry. Our data showed an increase in the percentage of apoptotic MHCC97H-siNS cells compared with the siNC group. In addition, the percentage of apoptotic MHCC97H-siNS cells treated with the caspase 3 inhibitor was decreased by more than 30% compared with MHCC97H-siNS cells in the absence of the caspase 3 inhibitor after UV irradiation. The percentage of apoptotic MHCC97H-siNS cells treated with the caspase 3 inhibitor was almost equal to that of apoptotic MHCC97H-siNC cells (Fig 6C–6E). Therefore, our results demonstrated that caspase 3 inhibition prevented apoptosis induced by knockdown of NS with UV treatment.

## Discussion

NS is a p53-binding protein that exists mainly in the nucleoli of embryonic and adult stem cells as well as various types of cancer cells, but not in committed or terminally differentiated cells [1]. Expression of NS disappears before changes in the cell cycle during the development of neural stem cells in vivo, indicating that disappearance of NS expression induces cell cycle arrest, but not the reverse. Moreover, silencing NS by siRNA in stem cells of the central nervous system and U2OS cancer cells increases the number of non-cycling and apoptotic cells. In addition to its role in the cell cycle, NS is related to ribosomal biosynthesis [8], senescence [17–19], and apoptosis [21–22, 33]. For example, suppression of NS increases apoptosis of cervical cancer cells and 5637 bladder carcinoma cells [21, 33].

Since the first report of NS in 2002 by Tsai et al, some studies have suggested that NS functions to maintain the proliferative capacity of stem and cancer cells [13, 15, 25]. However, the precise effects of NS vary in different cell lines. For example, in SW5637 cell lines, NS inhibition increases the numbers of apoptotic cells, whereas apoptosis is not enhanced in SW1710 cells [21]. Similarly, in most cell lines, knockdown of NS causes G0/G1 arrest, whereas G2/M arrest occurs in others [15–16]. Some studies have reported that NS plays an important role in the cell cycle and apoptosis of certain cancer cell types. Moreover, a study investigating NS and apoptosis-stimulating of p53 protein 2 (ASPP2) expression and their effect on pituitary adenoma cell proliferation suggested that NS may be a potential target for pituitary adenoma gene therapy [34].

HCC is a deadly disease with very high morbidity and mortality worldwide. However, the function of NS in HCC has not been explored. In our study, immunofluorescence showed that most NS protein was located in the nucleolus (S4 Fig), which is agreement with the study of McKay et al [1]. Real-time PCR and western blotting revealed higher NS expression in most HCC tissues than their matched adjacent tissues. In addition, most liver cancer cell lines had high levels of NS protein compared with the control, suggesting that NS may participate in tumor progression or apoptosis repression in HCC. Next, MTT assays showed obvious suppression of cell growth after NS knockdown for 24 h in MHCC97H and Bel7402 cell lines, which was supported by the results of cell proliferation assays. These data inferred that knockdown of NS decreases the cell viability of MHCC97H cells. Additionally, we found that NS suppression increased cell cycle arrest mainly at G2/M phase.

Targeting apoptosis pathways is an effective measure for cancer treatments [35, 36]. Previous studies of NS were mainly focused on its role in the cell cycle and rarely on apoptosis [37–38]. Our results showed a significant increase in the percentage of apoptotic cells after NS knockdown compared with the control following UV irradiation or serum starvation, whereas a significant decrease in apoptotic cells was induced by NS overexpression.

Caspase 3 is a crucial executer of apoptosis [39], whereas Bcl-2 is an important anti-apoptotic protein. Our study indicated higher expression of caspase 3 in the NS knockdown group than the control after UV irradiation or serum starvation, while Bcl-2 was lower in the NS knockdown group than the control group. In addition, a caspase 3 inhibitor obviously rescued NS suppression-induced apoptosis, suggesting that caspase 3 plays a pivotal role in NS-related apoptosis. Knockdown of NS could promote p53 expression [40], and p53 could control the expression of cleaved caspase 3 and decrease expression of Bcl2. So NS knockdown might be dependent on p53 to change the expression of caspase3 and Bcl2. Collectively, these data indicated that NS silencing by siRNA promoted apoptosis of HCC cells, including MHCC97H and Bel7402 cell lines, after UV irradiation or serum starvation.

In conclusion, our results suggest a regulatory role of NS in coordinating the proliferation and apoptosis of HCC cells. Most HCC cell lines and tissues exhibited high NS expression, and we observed a significant reduction in the proliferation of siNS-treated cells. However, despite the initial presumption that NS promotes proliferation, our study suggests an important role in apoptosis inhibition by NS in HCC cell lines. NS silencing-induced apoptosis was accompanied by up-regulation of caspase 3 and down-regulation of Bcl-2. Taken together, our findings have revealed the role of NS in HCC cell proliferation and apoptosis. Whether NS promotes the progression of HCC in vivo, and whether targeting NS is as a novel therapeutic approach for liver cancer will be worthy research topics for further studies.

## Supporting Information

S1 Fig HCC cell lines exhibit higher proliferation rates compared with L02 cells. The proliferation of HepG2, MHCC97H, SMMC7721, Bel7402, and L02 cell lines was detected by MTT assays. Each value represents the average of three independent experiments, and bars indicate the standard error of the mean.
(TIF)

S2 Fig Transfection with siNS or NS overexpression does not affect the proliferation rate of SMCC7721 cells. MTT assays of SMMC7721 cells with NS knockdown (A) or overexpression (B).
(TIF)

S3 Fig NS knockdown sensitizes HCC cells to serum starvation-induced apoptosis. Apoptosis was strongly regulated by NS knockdown in Bel7402 (A) and MHCC97H (B) cells. For serum starvation, the culture medium was replaced with serum-free medium after transfection for 24 h, and then the cells were cultured for appropriate times. (C) Total apoptotic cells including viable and nonviable apoptotic cells in Bel7402 and MHCC97H cell lines. Apoptosis is displayed as mean ± SD values. Each treatment was repeated in triplicate with NS knockdown was visibly higher than that of control group. **P<0.01.
(TIF)

S4 Fig Localization of NS in MHCC97H cells. (TIF)

## Abbreviations

DMEM: Dulbecco’s modified Eagle’s medium
FBS: fetal bovine serum
GNL3: guanine nucleotide binding protein-like 3
HCC: hepatocellular carcinoma
NS: nucleostemin
RT: reverse-transcription
siRNA: small interfering RNA
TRF1: telomeric repeat-binding factor 1
