# Supplementary figures and images for: Nucleostemin Knockdown Sensitizes Hepatocellular Carcinoma Cells to Ultraviolet and Serum Starvation-Induced Apoptosis
Source: PLoS One. 2015 Oct 30;10(10):e0141678. doi: 10.1371/journal.pone.0141678 (PMC4627730; doi:10.1371/journal.pone.0141678)

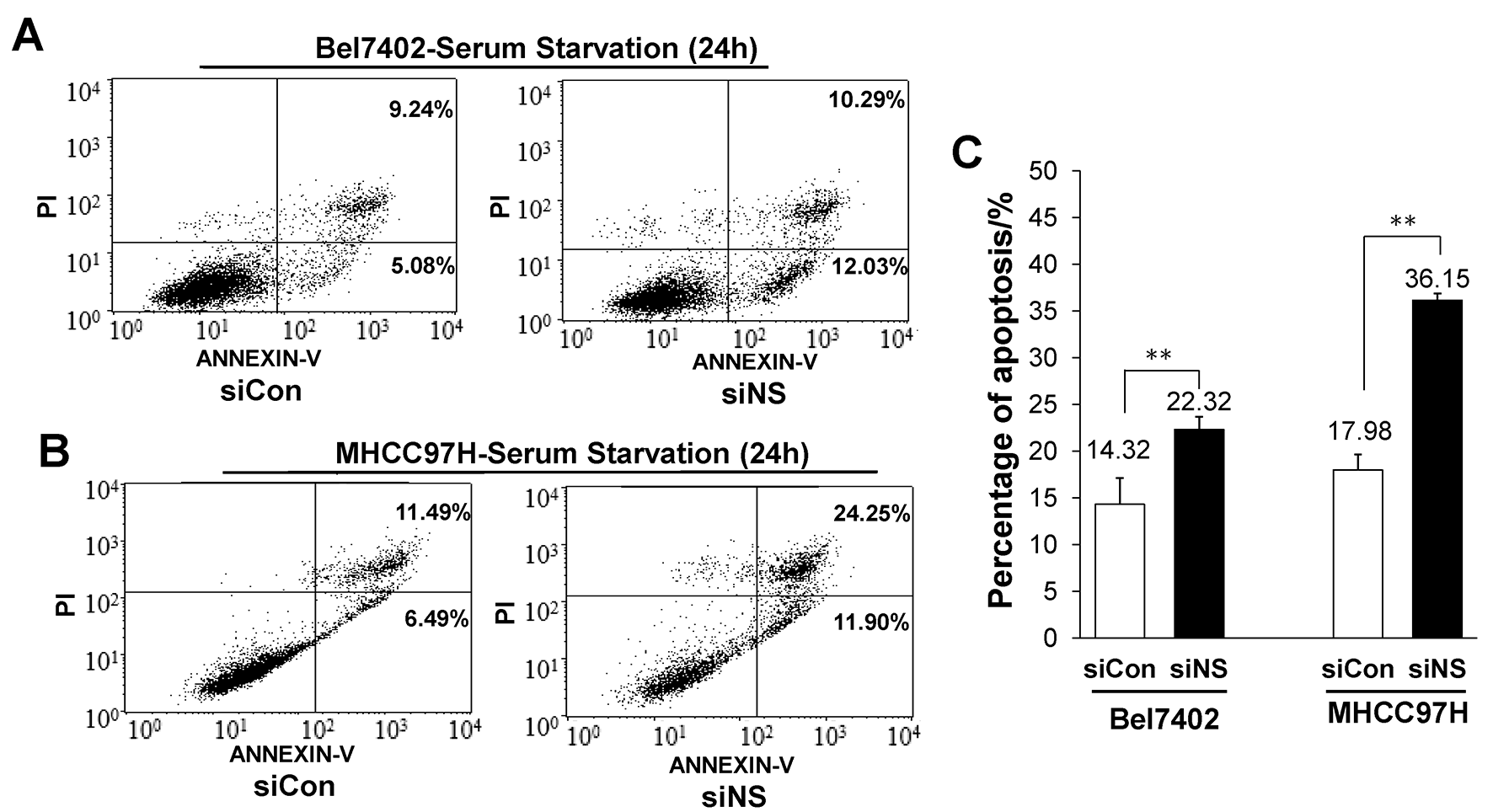

Supplement: S3 Fig — Apoptosis was strongly regulated by NS knockdown in Bel7402 (A) and MHCC97H (B) cells. For serum starvation, the culture medium was replaced with serum-free medium after transfection for 24 h, and then the cells were cultured for appropriate times. (C) Total apoptotic cells including viable and nonviable apoptotic cells in Bel7402 and MHCC97H cell lines. Apoptosis is displayed as mean ± SD values. Each treatment was repeated in triplicate with NS knockdown was visibly higher than that of control group. **P<0.01. (TIF) [file pone.0141678.s003.tif]

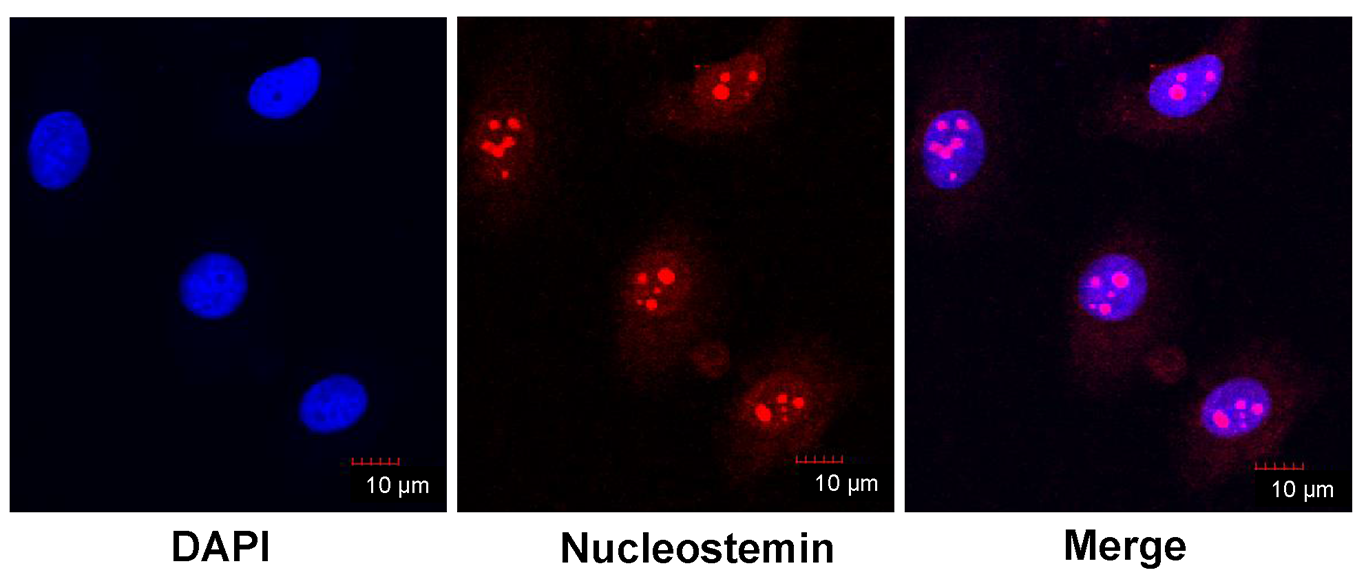

Supplement: S4 Fig — (TIF) [file pone.0141678.s004.tif]
